# Supplementary material for: Heparin Provides Antiviral Activity Against Rhinovirus-16 via an Heparan Sulfate Proteoglycan-Independent Mechanism
Source: Int J Mol Sci. 2025 Oct 25;26(21):10393. doi: 10.3390/ijms262110393 (PMC12608904; doi:10.3390/ijms262110393)
Supplement: Supplementary file 1 [file ijms-26-10393-s001.zip › ijms-3905886-supplementary.pdf]

Supplementary Materials

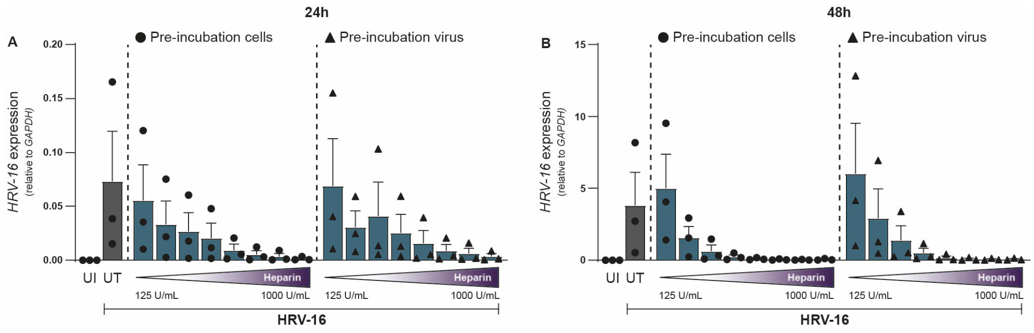

Supplementary Figure S1. *Antiviral effect of Heparin on HRV-16 Infection in H1-HeLa Cells (raw data). Experimental set-up and figure lay-out is similar to Figure 1 and HRV-16 RNA expression was quantified using RT-PCR. Uninfected (UI; white bar) and HRV-16 infected but untreated (UT; grey bar) conditions were taken along as control. HRV-16 RNA expression is relative to housekeeping gene GAPDH (A-B). The graphs represent collated data (mean ± SD) from 3 independent experiments using two replicates per experiment. A two-way ANOVA with Tukey's multiple-comparison test was performed for unpaired observations. Statistical significance was set at *P < 0.05, **P < 0.05, ***P < 0.005, ****P < 0.0005.*

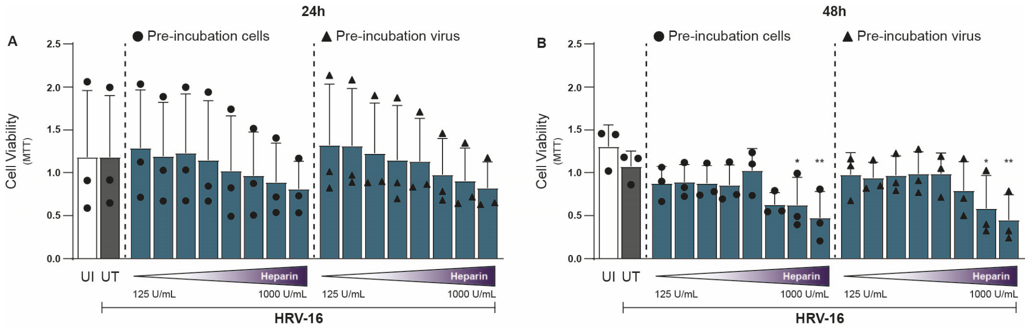

Supplementary Figure S2. *Heparin is tolerated by cells up to a level of 750 IU/mL at 48 hpi (raw data). Experimental set-up and figure lay-out is similar to Figure 2 and well viability was assessed using an MTT assay. Cell viability is indicated by OD580 (A-B). The graphs represent collated data (mean ± SD) from 3 independent experiments using two replicates per experiment. A two-way ANOVA with Tukey's multiple-comparison test was performed for unpaired observations. Statistical significance was set at *P < 0.05, **P < 0.05, ***P < 0.005, ****P < 0.0005.*

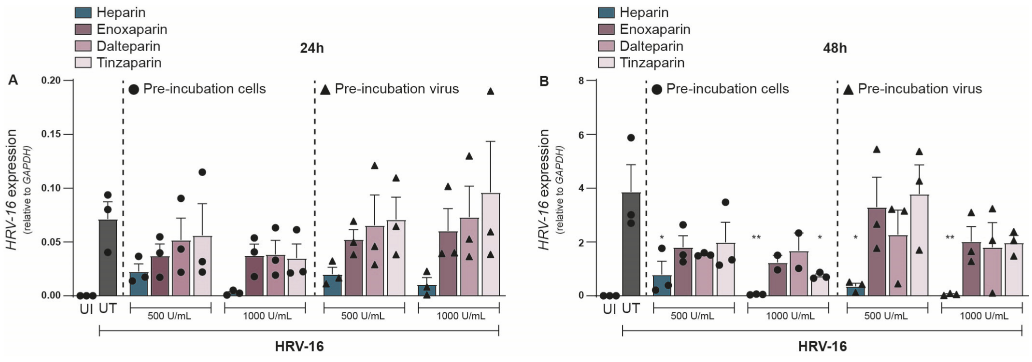

Supplementary Figure S3. *Experimental set-up and figure lay-out is similar to Figure 2 and HRV-16 RNA expression was quantified using RT-PCR. HRV-16 expression is relative to housekeeping gene GAPDH (A-B). The graphs represent*

*collated data (mean ± SD) from 3 independent experiments using two replicates per experiment. A two-way ANOVA with Tukey's multiple-comparison test was performed for unpaired observations. Statistical significance was set at *P < 0.05, **P < 0.05, ***P < 0.005, ****P < 0.0005.*
